# BACE1 at the crossroads of a vicious circle between Alzheimer’s disease and diabetes mellitus

**DOI:** 10.3389/frdem.2025.1730524

**Published:** 2025-12-09

**Authors:** Masuo Ohno

**Affiliations:** Center for Dementia Research, Nathan Kline Institute, Orangeburg, NY, United States

**Keywords:** Alzheimer’s disease, BACE1, insulin resistance, diabetes mellitus, amyloid-β, biomarker, cognitive impairment, precision medicine

## Abstract

Alzheimer’s disease (AD) and type 2 diabetes mellitus (DM), both of which are characterized by increased prevalence with aging, have considerable overlap in their risk factors, comorbidities and pathophysiological mechanisms including insulin resistance. While Alzheimer’s β-secretase BACE1 is primarily expressed in the brain, it is also present in peripheral tissues at lower levels. Interestingly, BACE1 not only initiates the sequential cleavage of amyloid precursor protein to generate amyloid-β (Aβ) peptides but also cleaves the ectodomain of insulin receptors. Given a growing body of research showing that increased Aβ and insulin resistance elevate BACE1 level/activity, BACE1 represents a key molecule that is situated at the crossroads of a vicious circle between AD and DM. Remarkably, BACE1 level/activity is found to increase under insulin resistance in type 2 DM patients and animal models, which may represent a contributing factor to the progression to AD. This review provides an overview of BACE1 mechanism as a dual disease-modifying therapeutic target to mitigate *β*-amyloidosis and insulin resistance that underlie cognitive decline at the intersection between AD and DM.

## Introduction

While Alzheimer’s disease (AD) is considered primarily as a disease of the central nervous system (CNS) representing the leading cause of dementia, it is accompanied by metabolic dysfunction and insulin resistance in the brain and peripheral tissues that have been long recognized as pivotal features of type 2 diabetes mellitus (DM) (Zhao and Townsend, 2009; Arnold et al., 2018; Rhea et al., 2022; Ezkurdia et al., 2023). AD is sometimes referred to as type 3 DM reflective of a brain form of diabetic conditions, given that insulin resistance occurs independently or overlaps with DM (de la Monte, 2019). A large body of epidemiological evidence indicates that type 2 DM, obesity and other prediabetic conditions of insulin resistance are risk factors for developing AD (Arnold et al., 2018). In particular, a large-scale longitudinal cohort study demonstrates that younger age at the onset of DM is associated with higher risk of subsequent diagnosis of AD and dementia (Barbiellini Amidei et al., 2021). Moreover, up to 81 percent of AD cases show either type 2 DM [fasting plasma glucose (FPG): ≥126 mg/dL] or prediabetes (FPG: 110–125 mg/dL) (Janson et al., 2004), suggesting that patients with AD are more susceptible to DM. Although the findings strongly suggest the crosstalk between AD and DM, the underlying molecular mechanisms including how deficient CNS and peripheral insulin signaling may be linked to AD pathophysiology remain elusive.

**Figure 1:**
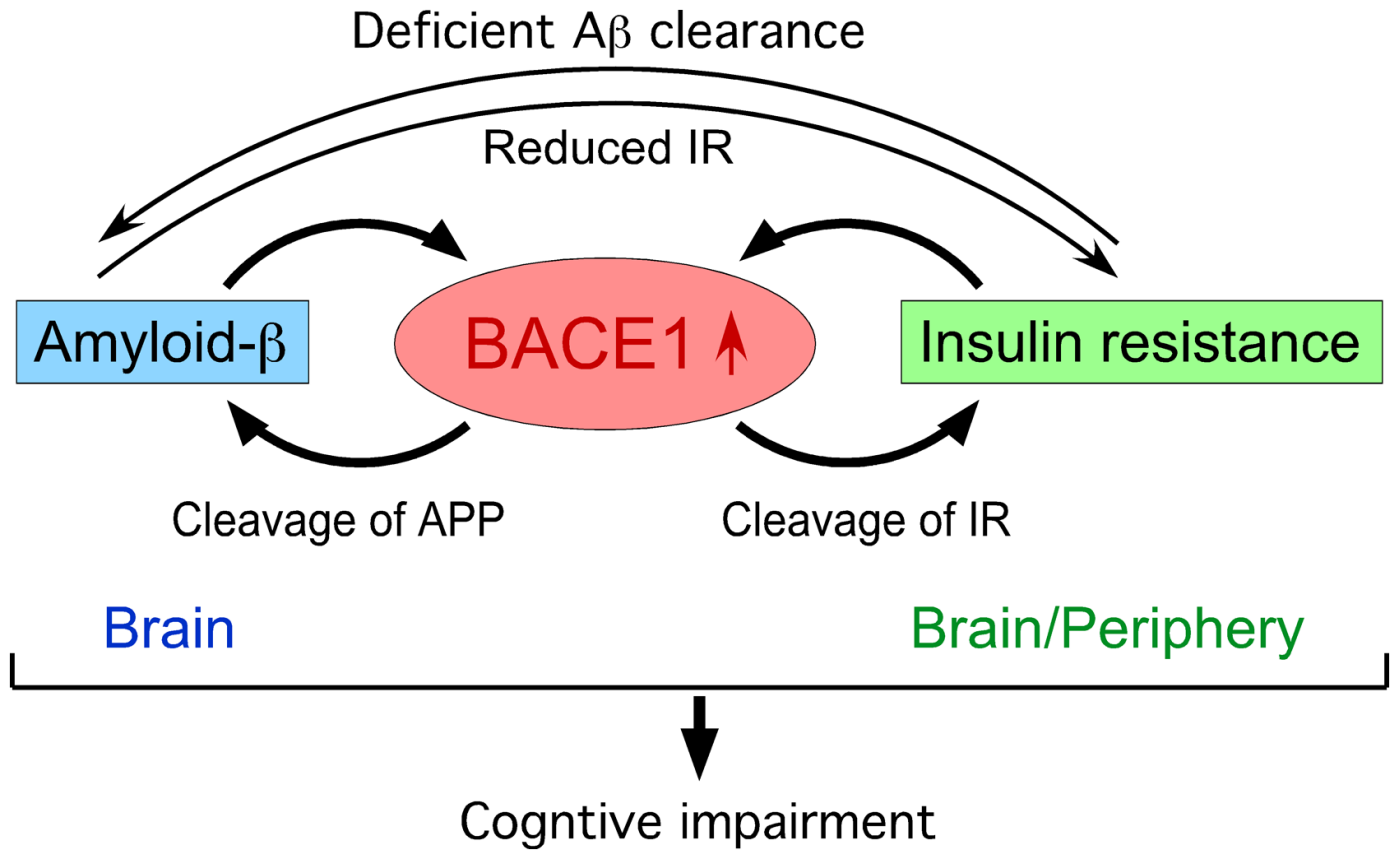
BACE1 at the crossroads of a vicious circle between amyloid-β and insulin resistance.

Over the recent decades, a multiplicity of investigations from basic, translational to clinical research have contributed to unveiling pathogenic roles of the Aβ pathway in driving other neuropathological hallmarks of AD that eventually lead to cognitive and clinical symptoms (Hampel et al., 2021a). The amyloid hypothesis is supported by the approval of the first disease-modifying therapies by passive immunization with anti-Aβ monoclonal antibodies such as lecanemab and donanemab, which significantly reduce brain Aβ levels and slow cognitive and clinical decline in early AD (Sims et al., 2023; van Dyck et al., 2023; Rafii and Aisen, 2025). The relationship between type 2 DM or insulin resistance and the degree of Aβ pathology in positron emission tomography (PET) imaging as well as post-mortem AD brains was largely negative when studies were conducted after the onset of cognitive symptoms (Arnold et al., 2018). It is well known that Aβ accumulation commences 15–20 years before the manifestation of obvious cognitive impairment in sporadic AD (Villemagne et al., 2013) as well as in genetic forms of AD such as dominantly inherited AD (Bateman et al., 2012) and Down syndrome (Fortea et al., 2020). Importantly, a recent longitudinal study in dementia-free individuals showed that DM diagnosed 7 years prior to PET assessment was linked to the higher degree of brain Aβ pathology, which correlated with increases in blood glucose concentrations (van Arendonk et al., 2023). In this review article, I will briefly summarize the literature reporting BACE1 mechanisms that may account for bidirectional interactions between insulin resistance and Aβ plaque growth in an early or preclinical stage of AD (Figure 1).

## BACE1 elevation associated with deficient insulin signaling

The β-secretase BACE1 is highly expressed in the CNS and responsible for the rate-limiting first step of sequential cleavage of amyloid precursor protein (APP) to produce Aβ peptides (Hampel et al., 2021b). High BACE1 expression is found in close proximity to Aβ plaques in human AD and APP transgenic mouse brains (Zhao et al., 2007; Devi and Ohno, 2013), while BACE1 and APP co-accumulated in peri-plaque dystrophic neurites function to generate Aβ, suggesting that local BACE1 elevation and *de novo* Aβ synthesis form a positive feedback loop to drive plaque growth (Zhang X-M. et al., 2009; Sadleir et al., 2016). Interestingly, clinical research indicates that serum BACE1 activity is significantly higher in individuals with DM as compared with healthy controls (Cervellati et al., 2022). While serum BACE1 activity is also higher in mild to moderate AD patients than age-matched cognitively unimpaired controls, there is no difference between diabetic versus nondiabetic individuals in the symptomatic phase (Cervellati et al., 2020). It is conceivable that diabetic states may be no longer a determinant of BACE1 activity in full-blown AD, consistent with no significant association between DM and PET Aβ burdens at this stage (Arnold et al., 2018). Notably, plasma BACE1 level and enzymatic activity elevated in patients with type 2 DM correlate with reductions in their cognitive scores (Bao et al., 2021). Another study also reveals a significant trend towards increased risk for mild cognitive impairment (MCI) with high plasma BACE1 level and insulin resistance in type 2 DM patients (Tian et al., 2020).

Animal model studies have been extensively performed to explore the mechanism of BACE1 as a risk for promoting cognitive impairment in DM patients through β-amyloidogenesis and insulin resistance (Figure 1). Although animal models are not individually a faithful reproduction of human DM or AD and have translational limitations, they provide useful tools to increase our understanding of molecular/cellular basis when the findings from different models are combined to address the experimental question of interest. High-fat diet (HFD) feeding followed by administration of low-dose streptozotocin (STZ), a well-characterized pancreatic islet β-cell toxin, recapitulates hyperglycemia associated with hyperinsulinemia and insulin resistance in mice and rats (Furman, 2021). These type 2 DM models show increased levels of Aβ and the β-secretase-cleaved C-terminal fragment of APP (β-CTF or C99, an intermediate β-metabolite of APP) concomitant with BACE1 elevations in the hippocampus and cerebral cortex, leading to learning and memory impairments (Zhang T. et al., 2009; Jiang et al., 2012). RNA interference-based reversal of increased BACE1 to control levels in the brain blocks DM-associated Aβ/β-CTF elevations and cognitive deficits in the STZ rat model (Yu et al., 2014). Furthermore, hippocampal BACE1 elevation also occurs along with cognitive impairment in an intracerebroventricular (ICV) STZ-injected mice (Santos et al., 2015), which reproduce features of sporadic AD including deficits in brain insulin signaling and energy metabolism, tau hyperphosphorylation, inflammation, neurodegeneration, among many others. Intranasal insulin restores reductions in insulin receptor (IR) expression and its downstream signaling in the hippocampus/cortex of ICV-STZ rats, which is accompanied by reversal of BACE1/A*β* elevations, impaired cognitive performances and other AD-like traits (Rajasekar et al., 2017). Together, the findings in STZ rodent models suggest that deficient hippocampal/cortical insulin signaling induces memory declines and Aβ/β-CTF overproduction due to increased BACE1 expression at the crossroads between AD and DM.

Young APP transgenic mice that develop little or no Aβ deposition yet in the brain have been used to test if/how experimentally induced diabetic conditions affect AD-like pathologies and cognitive deficits. STZ-treated diabetic APP/presenilin 1 (PS1) mice showed increased soluble human Aβ42 concentrations and plaque burdens in the hippocampus and cortex concomitant with deficient CNS insulin signaling (Wang et al., 2010). In this model, BACE1 elevation facilitated β-amyloidogenic processing of APP as measured by increased levels of β-CTF and the β-secretase-cleaved soluble ectodomain of APP (sAPP-β) and decreased sAPP-*α*, leading to aggravated learning and memory impairment. Similarly, BACE1 elevation-related exacerbation of AD-like phenotypes such as Aβ plaque deposition and impairments of cognitive function, synaptic plasticity and dendritic spine morphology has been reported in STZ-treated young 5XFAD mice (Devi et al., 2012), APP/PS1 mice fed long-term HFD during early life (Gong et al., 2021) and/or 3xTg-AD mice exposed to maternal HFD (Natale et al., 2023). Diabetic 5XFAD model reveals unfolded protein response (UPR)-associated activation of the PERK/eIF2α phosphorylation pathway (Devi et al., 2012), which is not only a key mediator of translational BACE1 upregulation in AD (Devi and Ohno, 2014; Ohno, 2014, 2018) but also suppressed by insulin application (Sullivan et al., 1999). The antidiabetic agent liraglutide, a glucagon-like peptide-1 receptor (GLP-1R) agonist, is shown to restore insulin signaling and reduce increased BACE1 activity, Aβ and tau hyperphosphorylation to control levels in insulin-resistant cells (Jantrapirom et al., 2020). Moreover, plasma GLP-1 levels decrease in APP23/PS45 mice and negatively correlate with brain Aβ load in AD patients, while an GLP-1R agonist improves cognitive impairment and lowers Aβ/β-CTF by reducing BACE1 expression in the hippocampus/cortex of APP23/PS45 mice (Zhang et al., 2025). This action is caused by inhibition of the transcription factor NF-κB-mediated promotion of *BACE1* gene following activation of the GLP-1R/AMPK pathway. Further investigation is needed to firmly establish a mechanistic link between impaired insulin/GLP-1 signaling and BACE1 elevation in the brain.

Other studies used obesity-dependent models of type 2 DM that derived from a mutation of the gene encoding leptin (ZDF rats) or its receptor (*db/db* mice), whose normal function precludes obesity by regulating food intake, glucose homeostasis and energy expenditure (Marwarha and Ghribi, 2012; Forny-Germano et al., 2019). Elevated levels of BACE1, Aβ and/or β-CTF were found in the hippocampus/brain of leptin-resistant *db/db* mice (Bonds et al., 2019) and ZDF rats (Lee et al., 2016) in parallel with their cognitive dysfunction and high blood glucose concentrations. Type 2 DM rats exposed to HFD and STZ treatment also showed hippocampal BACE1 elevation concomitant with deficient leptin and insulin signaling cascade, all of which were blocked by treadmill exercise, a protective factor against AD (Rezaei et al., 2023).

Intriguingly, both ZDF and STZ rat models of DM have increased levels of the early endosome marker Rab5 and Aβ/β-CTF in the hippocampus, while high glucose up-regulates Aβ level through Rab5-dependent endosome enlargement co-localized with β-CTF and BACE1 in cultured cells (Chae et al., 2020). Furthermore, age-dependent Aβ pathology is accelerated in brains of type 2 DM-affected cynomolgus monkeys compared with healthy controls, which is accompanied by enlarged early endosomes and APP accumulation in neurons as well as increased levels of Rab GTPases reflective of endocytic disturbances (Okabayashi et al., 2015; Kimura, 2019). These results are particularly of importance given that β-CTF-dependent endosomal-lysosomal dysfunction and enlargement are well characterized as an earliest AD pathological event in a multiplicity of mouse models and human AD-derived cells (Nixon, 2017; Bourgeois et al., 2018; Kwart et al., 2019; Pulina et al., 2020) and induce Aβ disposal failure inside neurons, eventually leading to extracellular plaque lesions (Lee et al., 2022; Im et al., 2023). Dysregulated BACE1-mediated APP processing for β-CTF/Aβ overproduction and related pathologies under deficient CNS insulin signaling on diabetic/obese conditions may be linked to AD progression in an early preclinical stage.

## BACE1-mediated induction of insulin resistance

Although BACE1 expression is highest in the brain, it is also found widely in peripheral tissues such as pancreatic β-cells, adipocytes and hepatocytes, where its elevated expression may cause metabolic disorders including DM and obesity via Aβ-independent processes (Taylor et al., 2022) (Figure 1). While IR is ubiquitously distributed in the periphery and CNS, it was recently identified as a novel BACE1 substrate in the liver (Meakin et al., 2018a). The cell surface expression of biologically active IR is regulated by the BACE1 cleavage of its ectodomain in a glucose concentration-dependent manner. Aberrantly upregulated BACE1 degradation of functional membrane IR in the liver and increased plasma soluble IR are observed in diabetic patients (Bao et al., 2021) as well as in *db/db* (Meakin et al., 2018a) and HFD (Lin et al., 2025) mouse models of type 2 DM, representing a mechanism of directly BACE1-mediated insulin resistance. Importantly, BACE1^−/−^ gene deletion (Meakin et al., 2012) and treatment with the BACE1 inhibitor elenbecestat (Lin et al., 2025) restore deficient IR and downstream signaling pathways in the liver of HFD-fed mice, leading to improved DM-related parameters including insulin sensitivity, glucose tolerance and cognitive function. Treatment with the BACE1 inhibitor LY2886721 also improves diabetic phenotypes in neuronal human BACE1 knock-in (PLB4) mice, a model for high comorbidity of DM and AD (Dekeryte et al., 2021). Moreover, BACE1 elevation occurs through the downregulation of microRNA (miR-6838-5p) in the adipose tissue of HFD-fed mice, while BACE1 suppression via miR-6838-5p overexpression can reverse insulin resistance and increases in blood glucose and body weight to normal levels (Han et al., 2025).

BACE1 is also reported to cause insulin/leptin resistance through Aβ-dependent mechanisms in the brain (Figure 1). HFD-induced BACE1 elevation and consequently accelerated Aβ42 production cause leptin resistance in the hypothalamus and aggravate body weight gain, whereas BACE1 inhibition or gene knockout can prevent these changes in obesity-associated DM model mice (Meakin et al., 2018b). Aβ oligomer-evoked hypothalamic neuron dysregulation is proposed to disrupt metabolic homeostasis and lead to insulin resistance and glucose intolerance, revealing a crosstalk between CNS and peripheral pathogenic mechanisms involved in high comorbidity of AD and DM in aging populations (Clarke et al., 2015). Mechanistically, soluble Aβ oligomers downregulate the surface expression of IR and cause neuronal insulin resistance (Townsend et al., 2007; Zhao et al., 2008), while insulin signaling functions to inhibit pathogenic binding of Aβ oligomers to protect synapses from their toxicity including IR loss (De Felice et al., 2009; Zhao et al., 2009). Therefore, a vicious circle may be formed between Aβ toxicity and IR impairment in the brain during AD progression (Zhao and Townsend, 2009; Moloney et al., 2010). Meanwhile, recent evidence highlights the unique role of BACE1 expressed in astrocytes that regulates Aβ clearance in the brain (Zhou et al., 2023). The study demonstrates that targeted astrocytic BACE1 deficiency blocks its cleavage of IR and facilitates downstream insulin signaling pathways, leading to increased Aβ uptake and degradation by reactive astrocytes.

## Discussion

The findings summarized in this review indicate that BACE1-mediated insulin resistance may be one of the key events situated at the intersection between AD and DM, although further investigation is needed to fully understand how this pathogenic mechanism may underlie the complex, bidirectional connections between the CNS and peripheral tissues (Arnold et al., 2018; Rhea et al., 2022). It is conceivable that BACE1 inhibitors may have dual beneficial mechanisms to halt or slow AD progression at the early stage not only by reducing brain Aβ production but also by directly alleviating insulin resistance in the CNS and periphery (Figure 1). While this review is mainly focused on BACE1, other approaches that target the mechanisms or pathways intersecting with insulin resistance and β-amyloidosis such as antidiabetic treatments are currently under preclinical and clinical investigations and have been reviewed in detail elsewhere (Corraliza-Gomez et al., 2025; Zheng et al., 2025).

Unfortunately, BACE1 inhibitors that have undergone advanced phases of clinical trials to date in mild-to-moderate and early/prodromal AD populations have been halted due to futility and/or side effects including signs of mild cognitive worsening at the higher dosage (McDade et al., 2021; Bazzari and Bazzari, 2022; Coimbra et al., 2024; Naidu et al., 2025). The failure of these trials may be due to too-high levels of BACE1 inhibition often targeted to reduce Aβ levels by >70% at the highest dose and too-late applications to AD stages manifesting cognitive symptoms with extensive Aβ accumulation (McDade et al., 2021; Ohno, 2024, 2025). Moreover, considering the little or no selectivity of all BACE1 inhibitors (up to ~3-fold over the close homolog BACE2) tested so far in phase 2/phase 3 clinical trials (McDade et al., 2021), we cannot completely rule out the possibility that their BACE2 cross-inhibition may contribute to side effects or reduce therapeutic benefits of BACE1 inhibition (Ohno, 2024, 2025). As such, the negative results of previous clinical trials do not necessarily exclude refined low-dose approaches with selective BACE1 inhibitors from the arsenal of preventive interventions at a preclinical stage of AD.

Given that multiple BACE1 substrates other than APP participate in complex physiological functions and pathways (Hampel et al., 2021b), potential problems in BACE1 inhibitor interventions including their on-target/off-target side effects have been extensively discussed (McDade et al., 2021; Coimbra et al., 2024; Naidu et al., 2025). In particular, excessively suppressed β-cleavage of synaptic BACE1 substrates such as seizure protein 6 (SEZ6) involved in maintaining spine dynamics (Zhu et al., 2018a; Zhu et al., 2018b) and close homolog of L1 (CHL1) related to axonal organization in adulthood (Ou-Yang et al., 2018; Vassar, 2019) may account for cognitive worsening observed with high-dose BACE1 inhibitors. However, neither partial BACE1^+/−^ gene reduction nor lower-dose BACE1 inhibitors that reduce Aβ up to 50% are free of adverse synaptic/cognitive effects, whereas cognitive worsening is unassociated with neurodegeneration and reversible shortly after withdrawal of BACE1 inhibitor treatment (Ohno, 2016; Zhu et al., 2018a; Satir et al., 2020; McDade et al., 2021; Pratsch et al., 2023; Tariot et al., 2024). The findings support the idea that a rational, low-dose approach targeting 25%–50% inhibition of BACE1 could offer safe and promising therapeutic interventions if initiated at the early preclinical stage of AD with monitoring of cognitive function and biomarkers relevant to efficacy and side effects (Ohno, 2024, 2025). This is supported by gene-based data demonstrating that the Icelandic APP mutation (A673T) that lowers Aβ by only 28% is protective against AD and age-related cognitive decline (Jonsson et al., 2012; Martiskainen et al., 2017).

Broad biological roles of BACE1 besides APP processing have been largely regarded as a weakness of BACE1-targeted AD therapy in safety. However, the discovery of IR as a new BACE1 substrate (Meakin et al., 2018a) reveals that BACE1-inhibiting strategies may have the advantage of alleviating peripheral insulin resistance and reducing DM-associated AD risks (Figure 1). This effect can be expected with low/safe doses of BACE1 inhibitors since they are more readily accessible to BACE1 in peripheral tissues compared with the CNS. Furthermore, BACE1 inhibitors exert dual beneficial effects on pathological progression in the brain at a preclinical AD stage by directly suppressing the A*β* pathway and by ameliorating insulin resistance through decreased BACE1 cleavage of cell-surface IR. BACE1 is validated as an early AD biomarker (Hampel et al., 2020), while a multiplicity of environmental and genetic risk factors for AD are shown to converge on increased BACE1 level/activity in animal models and clinical settings (Ohno, 2025). Given BACE1 elevation positioned at the intersection of a vicious cycle between Aβ accumulation and insulin resistance (Figure 1), BACE1 biomarkers are especially important under diabetic or prediabetic conditions and can be used in combination with other early AD biomarkers (Aisen et al., 2022; Rafii and Aisen, 2023) as well as sensitive cognitive measures (Ohno, 2023, 2024) to identify high-risk individuals who would benefit from preventive BACE1 inhibitor treatment. Further study is required to fully understand how DM affects the amyloid-tau-neurodegeneration (ATN) and cognition framework. Timely and effective management of DM during the early stage of subtle cognitive decline is crucial for slowing or preventing the progression to AD. Advances in this line of research would eventually lead to the development of precision medicine-oriented BACE1 inhibitor interventions in preclinical AD.
